# Practice of non-institutional delivery and its associated factors among women who gave birth in Southern Ethiopia, 2022

**DOI:** 10.1186/s12905-023-02683-8

**Published:** 2023-10-10

**Authors:** Temesgen Geta, Fekire Sugebo, Fekadu Anjulo

**Affiliations:** 1 School of Nursing, College of medicine and Health Science, Wolaita Sodo University, Wolaita, Ethiopia; 2 School of Midwifery, College of medicine and Health Science, Wachamo University, Hosana, Ethiopia

**Keywords:** Boloso Bombe, Woreda, Southern Ethiopia, Non-institutional delivery, Delivered women

## Abstract

**Background:**

Non-institutional delivery is one of the major reasons that results in high mortality rates for a mother and her neonate. The World Health Organization estimates that only 43% of mothers have access to skilled delivery services. A recent Ethiopian Mini Demographic Survey indicated that more than half of Ethiopian women have given birth non-institutionally. This shows that maternal health remains a major public health challenge in Ethiopia, irrespective of the government’s measures for institutional delivery. So, the aim of this study was to assess the practice of non-institutional delivery and its associated factors among women who gave birth in the study area.

**Methods:**

A community-based cross-sectional study was carried out on 260 study participants from June 1 to July 1, 2022, in Boloso Bombe Woreda. Data collection was done using a structured questionnaire, and systematic sampling techniques were used to select the study subjects. The data was entered into the EPI data version 3.1 and analyzed using SPSS version 25. The adjusted odds ratio, along with 95% confidence intervals, was used, and the level of statistical significance was declared at a P-value of 0.05.

**Result:**

Out of 260 women interviewed, 252 (97%) pregnant women participated in the interview. The prevalence of non-institutional delivery among study participants was 68.7% (95% CI: 63.1–72.9). Mothers who were a daily laborer [AOR = 6.6;95%CI(3.6(1.2–11.2), last pregnancy planned [AOR = 0.4; 95%CI (0.4(0.2–0.8)), an absence of antenatal care contacting history [AOR = 3.3; 95%CI (1.3–8.6)], respondents’ knowledge on the labor complication [AOR = 3; (95%CI); 3.5(2.2–6.1)], and place of first delivery [AOR = 8.7 95%CI(3.2–23)] were factors that significantly associated with practice of non-institutional delivery.

**Conclusion:**

This study indicated that the majority of study participants practiced non-institutional delivery in this study area. Thus, we strongly recommend that all responsible bodies take immediate action, such as community health education on pregnancy-related complications, encouraging ANC visits, and raising awareness of the advantages of preventing non-institutional delivery in order to reduce non-institutional pregnancy practices and improve the factors identified.

## Introduction

Globally, only 123 million women who give birth each year receive antenatal care, neonatal care, and delivery care. To promote maternal, neonatal, and children’s health, women need to access basic health care settings during delivery [1]. According to the World Health Organization (WHO), more than 90% of women in high-income countries give birth at health facilities, whereas in low-income countries, most women give birth outside a health center by untrained people [2]. Non-institutional delivery means just giving birth outside a health institution by an unskilled person [3].

Many women who give birth outside a health facility can face problems such as dizziness, a tender abdomen, blood loss, fetal death, and uterine rupture. These problems are often considered dangerous for women who give birth outside a health facility [4]. Most maternal mortality (MM) occurs during delivery and in the immediate postpartum period. All women should have access to basic maternity care during their pregnancy, such as clean and safe births. They should also have access to emergency obstetric care if necessary [5]. In 2017, as a result of non-institutional deliveries and other-related factors, 462/100,000 mothers and 11/100,000 newborns died in both developed and developing countries, respectively [6].

In addition, giving birth outside a health institution has a three-fold higher risk of developing complications and has a higher risk of death [7]. The rate of neurological dysfunction and seizure in non-institutional delivery is three times higher than in institutional delivery [8]. Increasing delivery in health institutions is a crucial approach to preventing the death of a mother and her baby. However, the practice of institutional delivery has not changed consistently to reduce maternal deaths in low and middle-income countries [9]. Although there have been some improvements in reducing maternal and infant mortality worldwide, countries in sub-Saharan Africa and South Asia accounted for 86 per cent of the global maternal mortality rate in 2017. Only Sub-Saharan African countries accounted for two-thirds of maternal mortality [4, 6].

The literature has identified several factors associated with the practice of non-institutional delivery. These include maternal educational experiences, occupation, previous antenatal care contacts, and knowledge of obstetric complications. However, the last pregnancy planned, and complications from previous deliveries were not assessed in previous studies. [4, 5, 7].

Ethiopia is one of the 15 countries that are in a very high alert or high alert and as being in a fragile state. The practice of institutional delivery is an essential approach to preventing 13–33% and 20–30% maternal and newborn mortality, respectively [10]. Although the Ethiopian Government and non-governmental organizations try to prevent women from giving birth outside health facilities, more than 50% of Ethiopian women give birth outside health care facilities [11]. This shows that our country is still facing challenges in non-institutional delivery [12]. In southern Ethiopia, few studies have been conducted on the scope and factors influencing the non-institutional delivery. The literature available focuses mainly on urban and institutional areas, with little information on updated rural areas. So, the aim of this community-based study is to assess the practice of non-institutional delivery among women delivered in southern Ethiopia.

## Methodology

### Study area, period and design

A community-based cross-sectional study carried out in Boloso Bombe Woreda from June 1 to July 1 2022. The district is located about 57 km from Wolaita, southern Ethiopia, and 435 km from Addis Abeba, the capital of Ethiopia. Boloso Bombe’s geographical location is 70 1’ 32’’- 70 11’ 30” N latitude and 370 26’ 18’’-370 39’’ 38’’ E longitudinal. According to the Boloso Bombe Woreda Basic Plan Report 2013Woreda has a total population of 114,342. Of these, 57,400 were women, 56,942 were men, and 26,642 were reproductive-age women. The district has 21Kebeles, one primary hospital, four health centers, and eight health posts.

### Population and eligibility criteria

Source populations are defined as all women who had given birth during the preceding six months and had resided in the district for at least a year. The study participants included of women who had given birth during the six months prior to the study and had resided for at least a year in the chosen region. Study participants were women who were selected and participated in the study during the data collecting period. The study did not include mothers who had any mental health issues, were deaf or difficulty of hearing, or both.

### Sample size determination and its procedures

A total of 260 sample sizes were determined using a single population proportion. 5% margin of error, 95% confidence interval, 19% non-institutional delivery from the previous study, and 10% non-response were considered to calculate the final sample size [13].

According to the World Health Organization recommendation, 30% of the seven Kebeles (small villages) were selected from the total Kebeles using lottery methods. The total number of mothers who delivered in selected kebeles between 2021 and 2022 was determined using immunization registers and health post family folders. The sample size of each kebeles was proportionally allocated to the total number of deliveries of each kebeles for the selected kebeles. The respondents were then recruited using a systematic sampling method. Every other respondent to each kebeles were interviewed.

### Study variables

The practice of non-institutional delivery was a dependent variable. Socio-demographic characteristics (age, educational status, occupational status), obstetric (parity, gravidity, ANC follow-up), and health care provider-related factors (privacy, respecting women, friendly behavior of service providers) were independent variables of the study.

### Operational definition

#### Non-utilization of institutional delivery

Deals with women who deliver the last baby outside a health facility by non-skilled or traditional attendants [14].

### Knowledge on labor complications

Knowledge level on labor complications measured using **s**ix major labor complications, such as failure to labor progress, fetal distress, malposition, fetal distress, excessive bleeding, and cephalopelvic disproportion. Those who mentioned greater than or equal to 3 labor complications were classified as having good knowledge [15]. And those who mention < 3 complications were assumed to have poor knowledge [15].

### Data collection and analysis

A structured questionnaire, including four parts, was used to collect data. These include socio-demographic information, factors related to women’s obstetrics, factors related to health providers, and knowledge of labour complications. The last component contains a question that contains a list of the six most important labor complications adapted from the previous study [15].

The data were collected through face-to-face interviews. 10%of the questionnaires were pre-tested in unselected kebeles before the actual data collection began. The data were collected by seven B.Sc. nurses and supervised by three health officers. One day training was provided to data collectors and supervisors on data collection procedures. Firstly, the questionnaire was written in English, translated by experts into the local language, and again translated into English to increase consistency. In order to maintain data quality, the data collectors were closely supervised by the supervisor before and during the data collection process. The principal investigator (PI) supervised the correct implementation of the procedure and checks the completeness and logical coherence of the data collection after collecting the data.

The completeness and coherence of the data have been checked, encoded and entered into EPI Data Version 3.1. To analyze it, it was exported to SPSS version 25. In order to present descriptive statistics, frequencies, percentages, mean, standard deviations, and tables were used. The crude odd ratio (COR) of 95% of the confidence interval was calculated using a bivariate logistic regression test to test correlation between the dependent and the independent and select a the candidate variables. Then the variables that were found to be P < 0.25 in the bivariate analysis are taken as candidates for the multivariable logistic regression. Finally, multivariable logistic regression with AOR was used to control possible errors and to identify associated factors of the prevalence of non-institutional services. A P value < 0.05 was considered statistically significant.

## Result

### Socio-demographic features

Of a total of 260 women interviewed, 252 (97%) fully responded to the interview. The average age of the study participants was 28.07 years, and the standard deviationwas5.3 years. The age range of the participants was between 15 and 29 years old.122 (48.4%) participants were housewives. 86% of them were married and 205(81.3%) completed secondary school (Table 1).

**Table 1:** Socio-demographic characteristics of the respondents at Boloso Bombe Woreda in Southern Ethiopia, 2022(n = 252)

Socio-demographic characteristics		Frequency	Percentage (%)
Respondents’ age	15–29	140	55.6
	30–40	83	32.9
	41–49	29	11.5
Respondents’ educational level	Cannot read and write	42	16.7
	Can read and write	59	23.4
	Primary school	39	15.9
	Secondary school and above	112	44.4
Respondents’ occupation	House wife	122	48.4
	Merchant	73	29
	Civil servant	37	14.7
	Daily laborer	20	7.9
Partners’ education	Cannot read and write	17	6.7
	Can read and write	76	30.2
	Primary school	64	25.4
	Secondary school and above	95	37.7
Partners’ occupation	Farmer	114	45.2
	Merchant	82	32.5
	Civil servant	45	17.9
	Others^**+**^	11	4.4
Respondents ethnicity	Wolaita	176	69.8
	Gamo-Gofa	22	8.2
	Kambata	41	16.3
	Amhara	11	4.3
	Others^**++**^	6	2.4
Respondents’ religious	Orthodox	69	27.4
	Muslim	56	22.2
	Protestants	78	31
	Others^**+++**^	49	19.4
Respondents’ marital status	Married	205	81.3
	Single	16	6.3
	Others^**++++**^	31	12.3
Decision maker	Women	145	57.5
	Husband	107	42.5
House hold head	Women	53	21
	Husband	199	79

+-Private employee, unemployed, Students, Daily laborer; ++-Oromo, Hadiya, Gurage,; +++-Apostolic, Catholic, Joba,; ++++-Widowed, Divorced

### Obstetric and provider-related factors among the respondents

This study showed that majority of the study participants (93.7%) had an abortion history of 0–3 times. Approximately 196 (77.8%) of the participants visited the ANC during their last pregnancy. Before birth, 198 women (76.6%) planned to have a pregnancy, with 42.9% having one–three children. 73 (21%) of the participants reported that they had not received care for respect during the previous delivery and 85 (34.5%) reported that their privacy has not been maintained during the previous delivery. The main reasons for non-institutional delivery were poor belief in health facilities, sudden onset of labor, and a lack of respect from health care providers (Table 2).

**Table 2:** Obstetric and health care provider related factors of the respondents at Boloso Bombe Woreda in Southern Ethiopia, 2022(n = 252)

Gravidity	1–3	127	50.4
	4–6	94	37.3
	> 6	31	12.3
Parity	1–3	108	42.9
	4–6	97	38.2
	> 6	47	18.7
Abortion	0–3	236	93.7
	>=4	16	6.3
ANC follow up	Yes	196	77.8
	No	56	22.2
Last pregnancy planned	Yes	198	78.6
	No	54	21.4
Place of previous delivery	Home	107	42.6
	Health institution	141	57.4
Intension to delivery place	Yes	173	68.7
	No	79	31.3
Labor complication of last pregnancy	Yes	140	55.6
	No	112	44.4
Decision maker on last delivery	Women and her husband	138	55.2
	Husband	107	41.7
	Women	6	2.4
	Health professional	2	0.8
Friendly behavior by provider	Yes	190	63.5
	No	62	36.5
Respectful care	Yes	179	79
	No	73	21
Privacy maintained	Yes	165	65.5
	No	85	34.5
Reason for non-institutional delivery	I have not seen any advantage from HI delivery	14	17.7
	Intentionally health profession cannot attend delivery	10	12.6
	Sudden onset of labour	13	16.4
	No respect from service provider	18	22.8
	Poor belief on institution	19	24.0
	Long distance	5	6.3

### Knowledge of the respondent on labor complications

**Fig. 1:**
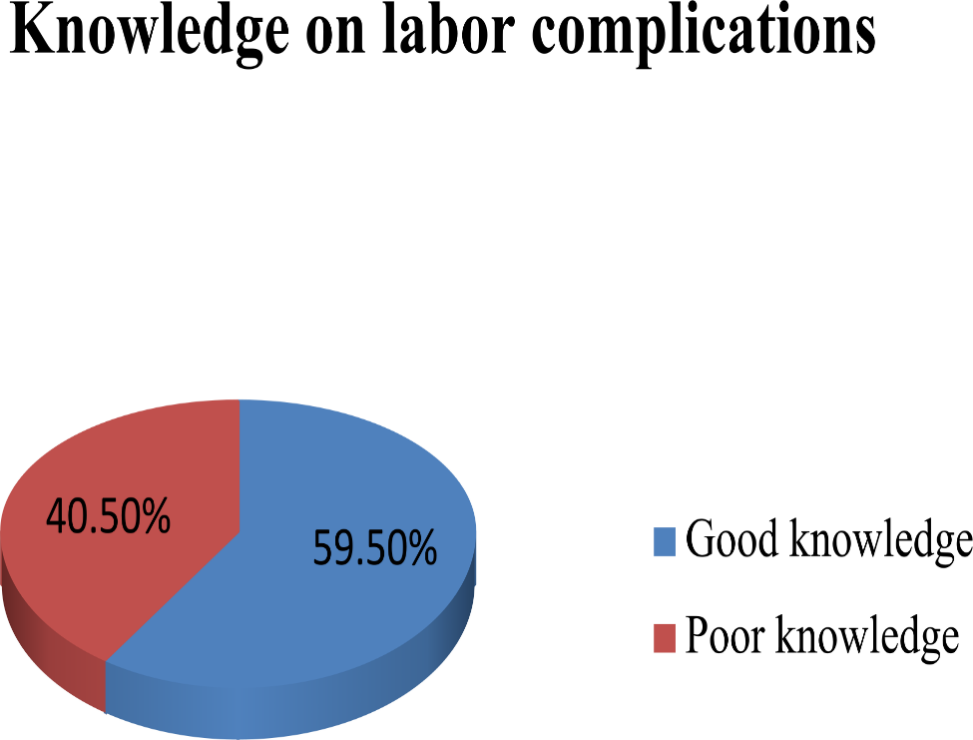
Participants’ knowledge on labor and delivery complications at Boloso Bombe Woreda in Southern Ethiopia, 2022(n = 252)

From the 252 participants, around 60% of women had poor knowledge of labor complications and 40% of them had good knowledge (Fig. 1).

### Prevalence of non-institutional delivery

**Fig. 2:**
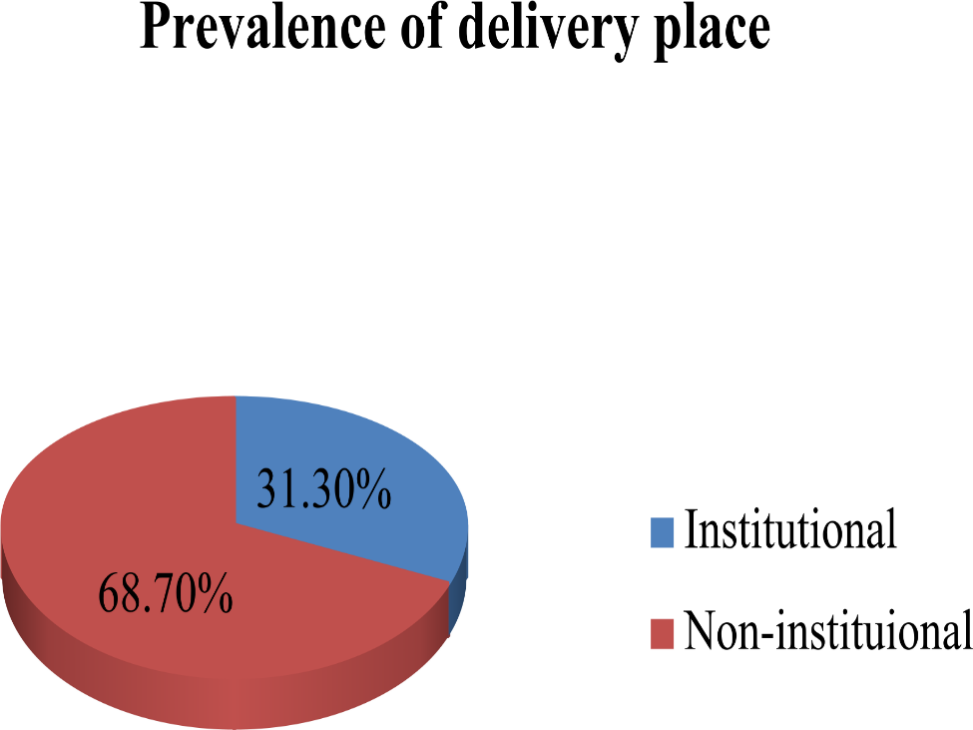
Place of last delivery among respondents at Boloso Bombe Woreda in Southern Ethiopia, 2022(n = 252)

Among the total study participants, 68.7% practiced non-institutional delivery, and 31.3% practiced institutional delivery (Fig. 2).

### Factors affecting the practice of non-institutional delivery

Bivariate logistic regression indicated that all variables, except place of first delivery, were significantly associated with dependent variables. Regarding multiple logistic regression, all variables selected for bivariate logistic regression were statistically associated with outcome variables. Mothers who were daily laborers were 6.6 times more likely to practice non-institutional delivery compared to their contrast group [AOR = 6.6; 95%CI (1.2–11.2)]. Women planning their last pregnancy are 60% less likely to give birth outside health institutions compared to unplanned women [AOR = 0.4; 95%CI (0.4 (0.2–0.8))]. Women who had no history of ANC follow-up were 3.3 times more likely to practice non-institution delivery compared to those who had an ANC follow-up history [AOR = 3.3; 95% CI (1.3–8.6)]. Participants who had poor knowledge of the labor complication were 3.5 times more likely to practice non-institutional delivery compared to their opposite group [AOR = 3.5; 95% CI (2.2–6.1)]. Women who gave birth outside of health institutions were 8.7 times more likely to do so than women who gave birth within health institutions [AOR = 8.7, 95% CI (3.2–23)] (Table 3).

**Table 3:** Factors associated with non-institutional delivery among respondents at Boloso Bombe Woreda in Southern Ethiopia, 2022(n = 252)

Factors		COR	p-value	AOR(CI)	p-value
Respondents’ occupation	Daily laborer	0.46(0.13–1.23)	0.27	3.6(1.2–11.2)	0.024
	Merchant	0.3(0.1–0.7)	0.11	4.3(1.4–13.1)	0.010
	House wife	0.2(0.1–0.69)	0.07	2.0(0.6–6.8)	0.26
	Civil servant	1	1	1	1
Last pregnancy planed	No	1.9(1.5–5.7)	0.08	0.4(0.2–0.8)	0.002
	Yes	1	1	1	1
Last pregnancy ANC follow	No	0.24(0.13–0.44)	0.00	3.3(1.3–8.6)	0.015
	Yes	1	1	1	1
Complication occurred at the last delivery	Yes	4(2.4–7.4)	0.00	2.5(1.2–5.2)	0.010
	No	1	1	1	1
Place of first delivery	Home	0.2(0.1–0.9)	0.21	8.7(3.2–23)	0.000
	Health post	2(1.2–6.2)	0.14	2.5(1.0–6.0)	0.000
	HC and hospital	1	1	1	1
Knowledge on obstetric complications	Poor Knowledge	0.4(0.2–0.72)	0.06	3.5(2.2–6.1)	0.011
	Good Knowledge	1	1	1	1

## Discussion

The non-institutional delivery practice in this study was 68.7%. This study is higher than the study done in the South Wollo Zone, Delanta district [16], Nepal (41.9%) [17], and Brazil (11.7%) [18]. Findings from the study are in line with the study done in Afar (71%) [19]. However, the variation in both cases may be due to differences in socio-demographic status, sample size, study period, geographic location, and methodological variation.

In this study, respondents’ occupational status was significantly associated with non-institutional delivery. Mothers who were daily laborers were three times more likely to give birth in non-institutions than mothers who were civil servants in occupation status. This study is supported by a study done in Gambela [14] and Benshangul Gumuz [20]. This may be due to the fact that being a daily laborer can inhibit them from getting health-related information easily, such as an advantage of institutional delivery and/or a disadvantage of non-institutional delivery; they may be easily exposed to economic problems that might inhibit access to a health facility; they are more exposed to family pressure and cultural influences. In addition, daily laborers had less awareness regarding complications of pregnancy, delivery, and labor, as well as the postnatal period, since they spent most of their time at work. Thus, this could increase the utilization of non-institutional delivery among them.

The place of previous delivery was significantly associated with non-institutional delivery. Women who gave their last birth outside a health center were 8.7 times more likely to give birth outside a health center than their counterparts. This study was in line with studies done in Ethiopia [21, 22]. The reason for this could be that mothers who lack adequate information about the benefits of institutional delivery may believe they are at lower risk of complications. As a result, this could increase the likelihood of non-institutional delivery and require close monitoring.

ANC is the most favorable period of contact for pregnant women to get adequate information about the risks and problems they may face during pregnancy, labor, delivery, and the postpartum period. The World Health Organization recommends that women without complications should have at least eight ANC contacts [23]. The study found that who had not followed were 3.3 times more likely to give birth outside a health center than those who had ANC follow-up. Which is consistent with studies done in the Delanta district [16], Zala Woreda [24] and, Nigeria [19]. This could be related to the fact that women who had no ANC might be less aware of birth preparedness and complications, danger signs of a pregnancy readiness plan, when to visit a health facility, and the danger of giving birth at a non-institutional place, which increases the chance of a non-institutional delivery.

Knowledge is an important factor that affects intentions, attitudes, and behavior. Lack of risk perception of delivery and labour could increase the use of non-institutional delivery [1, 22]. Study participants who had no previous knowledge of labor and delivery were 3.5 times more likely to give birth outside a health facility than their opposite group. The possible explanation might be that knowledge of labor and delivery complications is essential for early recognition of the problem and appropriate for timely utilization of institutional delivery services. Thus, women who do not have good knowledge of labor and delivery complications, tend to deliver at non-institutional places. Teaching mothers and the community about the complications of non-institutional delivery increases the need for a preference for place of delivery, which is likely to be more useful in contributing to decision-making.

Previous deliveries’ complications were identified as explanatory variables significantly associated with non-institutional delivery. Respondents who had complications were 2.5 times more likely to give birth outside of health facilities compared to their counterparts. The possible explanation might be the occurrence of complications that can contribute to stress and dissatisfaction and limit the utilization of institutional delivery. No previous literature indicated an association between complications occurring in previous health facilities and non-institutional deliveries [13–22]. Finally, the last pregnancy planned was negatively and significantly associated with the non-utilization of institutional delivery. Mothers who planned their last pregnancy were 60% less likely to practice non-institutional delivery compared to the opposite group. This may be because mothers who planned their last pregnancy may have an interest in seeking health care, following health care recommendations, and cooperating with their partners. No previous literature indicated an association between the last pregnancy plan and non-institutional delivery practice [13–22]. The main strength of the study was that, being community-based; it could reflect the experiences of the women during the study period. The limitation of the study was that it included mothers who gave birth one year prior to the survey, which might result in a recall bias.

## Conclusion

In general, 68.7% of mothers have given birth outside a health care facility. The main reasons for this are poor belief in the health care provider and the sudden onset of labor. Respondents’ occupation, last pregnancy planned, place of previous delivery, ANC follow-up, and knowledge of labor and delivery complications were associated factors. So, we strongly recommend that all stakeholders, such as health extension workers, health care providers, the district health office, and the district city administration, take immediate strategic actions to reduce the prevalence of non-institutional delivery and work more on the main reasons and factors that increase the practice of non-institutional delivery. Furthermore, actions aimed at maternal health education, encouraging ANC visits for pregnancy planning, and raising awareness about labor and delivery complications were critical to address for women giving birth outside of health facilities.

## Data Availability

The data collected and/or analyzed in the current study are not available to the public before publication to prevent any misuse by the public, but are available upon reasonable request from the corresponding author.
